# Cost-effectiveness of Adolescent Bariatric Surgery

**DOI:** 10.7759/cureus.248

**Published:** 2015-02-04

**Authors:** Sigrid Bairdain, Mihail Samnaliev

**Affiliations:** 1 Department of Surgery, Boston Children's Hospital; 2 Clinical Research Center, Boston Children's Hospital

**Keywords:** obesity, cost-effectiveness, health-related quality of life, quality-adjusted life-years (qalys)

## Abstract

Background: The current estimates of the prevalence of adolescent morbid obesity and severe morbid obesity are about 21% and 6.6%, respectively. Obesity, if left untreated, may result in a variety of comorbid conditions and earlier mortality. Adolescent bariatric surgery is an effective, but expensive means to ameliorate these conditions and the risk of earlier mortality. We aimed to develop a model to evaluate the long-term cost-effectiveness of bariatric surgery.

Methods: All adolescents who participated in our bariatric surgery multidisciplinary program from January 2010 to December 2013 were included if they had at least 12 months follow-up after their surgery. Intervention costs included all operative as well as preoperative and 12-month postoperative care. We used the US Medical Expenditures Panel Survey (MEPS) to estimate the association between reductions in BMI after surgery with future savings from reduced medical care use and with increased health-related quality of life (HRQL). We linked BMI with life expectancy using data from the Centers for Disease Control and Prevention. A Markov cohort model was then used to project health care-related costs (2013 US$), and quality-adjusted life years (QALYs) over time starting at age 18. Incremental costs per QALY of surgery vs. no surgery from a health care system perspective were then estimated.

Results: At one year follow-up, mean weight loss was 37.5 (std. = 13.5) kg and the corresponding BMI was 35.4 (reduction of 13.2, p<0.01). Mean total intervention costs/person were $25,854 (std. = 2,044). A unit change in BMI was associated with future medical care savings of $157/year (p<0.01) and with an increase in HRQL of 0.004 (p<0.01) and life expectancy. At a threshold of a 100,000/QALY, bariatric surgery was not cost-effective in the first three years, but became cost-effective after that ($80,065/QALY in year four and $36,570/QALY in year seven).

Conclusion: Our results suggest that bariatric surgery among adolescents may be cost-effective when evaluated over a long period of time. Future studies on a large scale are needed to show a continued improvement in QALYs and to evaluate earlier cost-effectiveness of the procedure.

## Introduction

The current estimates of the prevalence of adolescent morbid obesity and severe morbid obesity are 21% and 6.6%, respectively [1]. The burden of obesity on society is enormous; one study suggests that about $147 billion or 9% of all medical costs in the US in 2008 were obesity-related [2]. Much of this burden is driven by a number of co-morbidities, which occur at higher prevalence among obese patients, including diabetes, hypertension, sleep apnea, and depression [2]. Obesity may also result in earlier mortality if left untreated [3]. Bariatric surgery is an effective, although expensive, means to ameliorating obesity. Continued attention and resources have been allocated toward environmental changes and various public health policies to help combat the obesity epidemic; only in the last decade has there been a shift towards medical and operative interventions. Adult patients who undergo weight-loss surgery (WLS) live longer; the same conclusions have yet to be applied to the pediatric population [3]. Cost-effectiveness models have more recently been employed in adult counterparts to compare improvements following WLS in both disability-adjusted life years (DALYS) and quality-adjusted life years (QALYS). According to a recent NIH study, both gastric bypasses as well as gastric bands were cost-effective at a set level of $25,000/QALY compared to medical management of obesity [4]. These types of analyses have not routinely been applied to the pediatric population. As recent studies have documented that adolescents have experienced the same dramatic increases in obesity as seen in the adult population, we aimed to evaluate the long-term cost-effectiveness of bariatric surgery in this population [5].

## Materials and methods

This study was conducted with the approval of the Institutional Review Board at Boston Children’s Hospital (BCH) (IRB-P00007528). All adolescents who participated in our bariatric surgery multidisciplinary program from January 2010 to December of 2013 were included in the dataset and queried through retrospective review of their medical records. Patients were excluded if they did not have at least 12 months of follow-up following their surgery.

### Model

A Markov cohort model was used to project costs, body mass index (BMI), health-related quality of life (HRQL), and quality-adjusted life years (QALYs) over time, starting at age 18, and to compare these between two cohorts. The first cohort included patients who underwent bariatric surgery and the second included patients without bariatric surgery. The model consisted of yearly cycles and two health states: ‘alive’ and ‘dead.’ Changes in costs and QALYs between the cohorts were driven by the underlying difference in BMI, which in turn was linked with HRQL, life expectancy, and costs within each model cycle (year). We did not include separate health states for specific obesity-related comorbidities, because their impact on costs and QALYs are implicitly accounted for in the ‘alive’ state of our model. Specifically, individuals in the ‘surgery’ cohort can have higher HRQL and lower medical care costs compared to the 'no surgery' cohort, driven by their lower BMI, fewer associated comorbidities among individuals within that BMI, or both. In the 'surgery' cohort, we estimated reductions in BMI after surgery and assumed post-surgical weight regain of 5% per year in the first five years, plateauing at 25% after year five. In the ‘no surgery’ cohort, we assumed that BMI remained unchanged over time (BMI of 48.7).

### Input parameters

Estimation of cost-effectiveness requires three main parameters: added costs (one-time intervention costs net of savings from reduced medical care in the future), health related quality of life (HRQL), and probability of death within each yearly cycle. Our analyses adopted a health care system perspective. Charges associated with bariatric surgery were obtained from BCH accounting and included all preoperative, operative, and 12-month postoperative care, as well as overhead charges which, in BCH, are allocated to individual procedures and services. Charges were converted to costs using the 2013 cost-to-charge ratio (0.545) at our institution.

We linked BMI with annual total health care expenditures from the nationally representative Medical Expenditures Panel Survey (MEPS) to estimate savings from reduced medical care use associated with lower BMI after surgery [6]. MEPS contains total health care costs for all care provided in a sample of about 30,000 individuals during each calendar year. These include all out-of-pocket payments, and payments by private insurance, Medicaid, Medicare, and other sources. Using a linear regression model, we estimated change in these costs per unit of BMI. As this estimate is somewhat dependent on model specifications in sensitivity analyses, we used a range of values (0 to 100%) around the point estimate derived from MEPS [6].

We also used MEPS to estimate the association between BMI and health-related quality of life (HRQL) derived from questions from the EQ-5D survey. This estimate (0.0042, p<0.01) -- which was age- and gender-adjusted and limited to a subgroup of MEPS respondents with severe obesity (BMI ≥34) to more closely reflect our target population -- was lower compared to estimates by others, and therefore viewed to provide an upper bound estimate of cost-effectiveness with respect to that parameter. In sensitivity analyses, we also estimated cost-effectiveness using HRQL changes by BMI from other studies. We used life tables available from the CDC that estimate the probability of death within a year by age and BMI [6-7].

### Incremental cost-effectiveness ratio (ICER)

Incremental costs and quality-adjusted life years (QALYs) of surgery (vs. no surgery) were estimated over different time periods, discounting both costs and QALYs at 3%. We then estimated ICERs, which represented the costs of gaining an additional QALY. As bariatric surgery is expected to be more costly and more effective than no surgery, it can be considered cost-effective or ‘good value for money’ if it costs less than the societal willingness to pay for an additional QALY. We were guided by recent estimates of around $100,000/QALY of the societal willingness in the US to pay for an additional QALY, although cost-effectiveness at other thresholds is also presented [8].  

### Sensitivity analyses

We conducted probabilistic sensitivity analyses (PSA) with 1,000 bootstrap replicates to assess the robustness of our results to input parameter uncertainty. The PSA were based on a gamma distribution for intervention costs, medical cost savings, post-surgical weight loss, and a beta distribution for the HRQL gain associated with BMI reductions. In one-way sensitivity analyses, ICERs were recalculated when individual parameters were varied, including the gain in HRQL per unit of BMI, the intervention costs, the annual medical cost savings per BMI, the discount rate, and weight regain over time.

## Results

From January 2010 to December 2013, data from 11 patients were analyzed. Ninety percent (n=10) were female. Median age at surgery was 17 (1.3) years. Median preoperative body mass index (BMI) was 48.7 (6.6) kg/m^2^. All patients underwent a laparoscopic Roux-en-Y gastric bypass (RYGB) and 45% (n=5) had a concomitant hiatal hernia repair. Median length of stay was three days (range: 2-4 days). There were no perioperative or postoperative complications. Descriptive characteristics of these bariatric surgery patients are shown in Table 1.

**Table 1:** Descriptive characteristics of bariatric surgery patients (N=11)

Clinical Variable	Estimate
Female, n (%)	10 (90.9%)
Age, mean (SD)	17.3 (1.7)
Age, range	14 – 20
Height, mean cm (SD)	169.3 (8.8)
Weight, mean kg (SD)	138.2 (16.9)
BMI before surgery, mean (SD)	48.7(6.6)
Performed LRYGB, n (%)	11 (100%)
LRYGB and HHR, n (%)	5 (45.5%)
Length of stay, median days (range)	3 (2-4)
Complications, n (%)	0 (0%)

Intervention charges and costs are shown in Table 2 below. Preoperative care accounted for $2,081 in charges, mostly driven by the cost of diagnosis ($1,578). As expected, perioperative care accounted for the majority of charges ($42,918), mainly driven by the charges associated for surgery and related procedures ($29,036). Postoperative care charges ($2,439) were driven by clinic and ancillary charges. After adjusting for a cost-to-charge ratio of 0.545, mean total intervention costs/person were $25,854 (std. = 2,044). A unit change in BMI was associated with future medical care savings of $157/year. Table 2 also shows other input parameters based on our analyses. At one year follow-up, mean weight loss was 37.5 (13.5) kg and the corresponding BMI was 35.4 (reduction of 13.2, p<0.01). From MEPS, we estimated that a unit of BMI was associated with an increase in HRQL of 0.004. In addition, from data available from the CDC, a unit of BMI was associated with an increase in life expectancy, which was also associated with age [7].

**Table 2:** Input parameters for cost-effectiveness model for bariatric surgery

Parameter (Charges (2013 US$)	Base case
Pre-operative charges, mean (SD)	
Clinic	$339 (163)
Ancillary	$164 (89)
Diagnostic	$1,578 (1,061)
Perioperative charges, mean (SD)	
Ancillary	$113 (378)
Diagnostic	$944 (359)
Gen nursing	$6,498 (1,444)
Minimally invasive procedures	$13 (41)
Pharmacy	$3,002 (520)
Surgery and procedures	$29,036 (2,547)
Surgical foundation charges, mean (SD)	$3,312 (552)
Post-operative charges, mean (SD)	
Clinic	$633 (619)
Ancillary	$1,630 (1,134)
Surgery and procedures	$175 (584)
Total charges, mean (SD)	$47,438 (3,750)
Cost-to-charge ratio	0.545
Total Intervention costs, mean (SD)	$25,854 (2,044)
Annual savings per unit of BMI (standard error)^1^	-$157 (47)
HRQL gain per BMI (standard error)^1^	0.0042 (0.0011)
Weight loss after surgery, mean kg (SD)	37.5 (13.5)
Assumed weight regain post-surgery	5% / year (years 2 to 5)
Baseline annual probability of death at age 18 ^2^	0.008
Discount rate	3%

**Figure 1:**
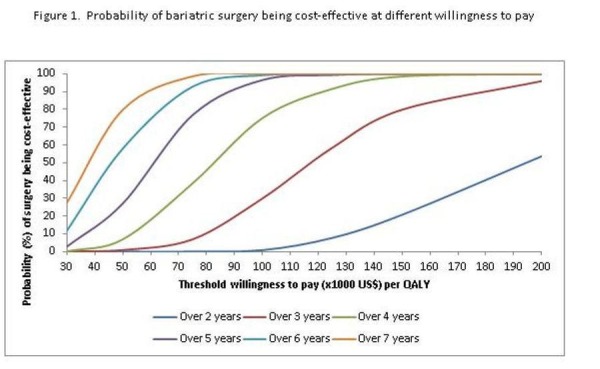
Willingness to pay thresholds The probability of surgery being cost effective at different willingness to pay (WTP) thresholds and time periods is shown in Figure 1.

Table 3 (below) presents the differences in overall health care costs, life expectancy, and QALYs based on the Markov model over the first seven years. (Longer projections did not change our conclusions.) Overall, bariatric surgery remained more expensive than no surgery in the first seven years, with increasing medical cost savings over time ($1,989 after the first year and increasing to $11,012 over seven years) which partially offset the intervention costs. Surgery was also associated with gains in life expectancy of around 0.006 years (2.2 days) over a year, and increasing to 0.158 years (57 days) over seven years, as well as with increased QALYs of about 0.06 and 0.457 after one and seven years, respectively. Based on a threshold of $100,000/QALY, bariatric surgery was not cost-effective in the first three years after surgery, but became cost-effective following that time interval. The probability of surgery being cost-effective at different WTP thresholds and time periods is shown in Figure 1 below. At $100k/QALY, this probability was about 75% over four years and greater afterwards. None of the patients suffered complications within the first year.

**Table 3:** Per person costs, additional years of life, and QALYs associated with bariatric surgery

Time horizon	Medical cost savings	Added life years	Added QALYs	Cost/QALY	95%CI	Cost-effective at 100k/QALY
Intervention cost = $25,854						
1 year	-$1,989	0.00624	0.060	$409,110	$235,034-$908,172	<O.5%
2 years	-$3,857	0.01791	0.123	$187,397	$110,031-$372,076	0.8%
3 years	-$5,564	0.03523	0.187	$115,304	$58,692-$209,870	29.9%
4 years	-$7,117	0.05810	0.252	$80,065	$43,452-$144,347	75.1%
5 years	-$8,523	0.08635	0.319	$59,474	$28,952-$105,818	96.5%
6 years	-$9,792	0.11978	0.386	$46,157	$18,991-$87,361	99.2%
7 years	-$11,012	0.15815	0.457	$36,570	$13,894-$71,255	99.9%

### Other sensitivity analyses

Our results were most sensitive to the underlying change in HRQL by BMI. As mentioned above, our estimates were based on the association between BMI and HRQL derived from MEPS. In one-way sensitivity analyses, ICER after four years varied from $2,700/QALY to $469,333/QALY (assuming no impact of BMI on HRQL). Under the latter assumption of no impact on HRQL, surgery became cost-effective after nine years (ICER of $80,724/QALY).

Results were also sensitive to the intervention costs when these costs were varied from $12,927 (half) to $51,708 (double of the point estimate). ICER over four years ranged from $24,828/QALY to $190,540. It is worth noting that costs at BCH may be greater than the US average, which would make our base case estimates conservative. When the medical care costs savings were varied from $0 to $314 per unit of BMI, ICER ranged from $110,922/QALY to $49,208/QALY, respectively. When the discount rate was varied, ICER (after four years) changed from $76,191/QALY (at 0% for both costs and QALYs) to $88,128/QALY (at 6%). Finally, the base case analyses assumed weight regain of 5% per year in the first five years. Our estimates over a period of five years changed from $51,719/QALY to $93,185/QALY when this was varied from no weight regain to 20% regain per year (complete weight regain after five years), respectively.

## Discussion

This study aimed to develop a model to evaluate the long-term cost-effectiveness of bariatric surgery among our cohort of adolescents. Whereas it has been shown that adults who undergo WLS live longer, the same conclusions have yet to be applied to the pediatric and adolescent population [3]. Despite the need for novel and equitable approaches to obesity, Oyetunji, et al. (2012) remarked that approximately 2% of morbidly obese children with a major comorbidity underwent a bariatric procedure [5]. This may be due to a combination of factors, including the necessity for centers to perform a higher volume of pediatric bariatric surgery cases before accreditation; the initial cost and factors altering the ability to be reimbursed per state; as well as the public’s concern for permanent procedures to be performed in either children or adolescents. In this study, we provide evidence, through an economic model, that the application of WLS to our cohort of adolescents was associated with gains in life expectancy, quality of life, and was cost-effective over long term, albeit, using a small sample size.

Overall, the idea of cost-effectiveness of bariatric surgery is not new; yet its application in those patients < 18 years of age has been somewhat limited by an overall lack of prospective, long-term data. In the adult realm, there is more long-term data regarding both improvement in quality of life, as well as less overall health care utilization following weight loss surgery (WLS). For example, Christou, et al. (2004) examined the health care utilization of both morbidly obese and obese patients for a total of seven years following their bariatric/weight loss operation; they observed significant decreases in comorbid conditions, mortality, and readmissions to the hospital for associated health conditions [9]. This is consistent with our model that also suggests reduced mortality, as well as medical care utilization. Similar to our study, Picot, et al. (2009) reported that bariatric surgery for obesity (BMI > 30) was cost-effective with a slight decrease in cost-effectiveness at lower ranges of BMI (30-35) [10]. Thus, there should be an impetus for weight loss surgery applied to appropriate BMI brackets and that more intensive medical regimens may be warranted for smaller BMI brackets from a cost-effectiveness perspective.

According to Aikenhead, et al. (2011), their meta-analysis of bariatric surgery also showed that bariatric surgery was a useful tool in older children given the reduction in associated comorbidities, as well as quality of life concerns. However, they warned that given the retrospective nature of most studies and permanent nature of some of the weight loss operations, larger studies and “less permanent/reversible” operations should be employed [11]. We acknowledge that our cohort was slightly older but that was related to some of the initial requirements for our program, including sustained weight loss preoperatively and clearance from our social workers and psychologists, as well as being a new program. We also acknowledge that not all bariatric operations are the same and that the best outcomes operation, as well as the safest operation, should be employed per patient. One of the few studies examining the comparison between medical interventions and surgical intervention in children and adolescents reveals that the interventions with the greatest health and quality of life benefits are laparoscopic adjustable gastric banding, reduction in television advertisements of high-sugar/fat food and drinks, as well as multi-faceted school programs for increased physical activity [12]. While we recognize that surgery plays an overall important long-term role, we do not negate the importance and value of continued attention to reduction of high-fat and high-sugar foods, as well as increasing physical activity. All of these factors play an integral role in long-term, successful weight loss, especially when it relates to adolescents’ longevity.

Future evaluations are warranted to compare the cost-effectiveness of bariatric surgery versus various lifestyle interventions. While we have alluded to this previously, obesity is often measured as its associated comorbid conditions' costs. The most prevalent is diabetes, as it is a public health dilemma in both the adult and pediatric realm. Contemporary adult studies have shown that bariatric surgery may increase quality of life, mitigate complications at a lower lifetime cost, and decrease associated morbidity and mortality [13-16]. While we did not look specifically at the cost-reduction as it related to diabetes and other comorbid conditions in particular, anecdotally, all 11 cases had reversal of their major comorbid conditions. Future prospective studies are needed to ascertain the reduction in costs seen following introduction of this surgery in this population.

### Limitations and strengths

One limitation is that while we had a precise estimate of the costs of bariatric surgery at our institution, we relied on some external data for HRQL, life expectancy and savings. Future evaluations will measure and incorporate the change in HRQL from pre- to post-surgery in our adolescent population. Another limitation is that our model did not distinguish between the effects of BMI vs. specific comorbidities on costs and QALYs; however, studies that have taken that approach have been restricted to fewer comorbidities and have likely underestimated the overall impact of obesity. The advantage of our approach is that, in principle, it captures the impact of all obesity-related comorbidities and complications. Disadvantages include the inability to disentangle the independent effect of BMI vs. specific comorbidities on our outcomes. On the other hand, one of the main strengths of this study is the long-term evaluation of bariatric surgery and the use of sensitivity analyses. It is encouraging that our findings remained relatively robust in these analyses, particularly those involving the link between BMI and HRQL and the intervention costs; these results were most sensitive.

## Conclusions

In conclusion, the application of bariatric surgery may be both a useful medical adjunct, as well as a cost-effective means of achieving permanent weight loss and providing resolution of comorbid medical conditions.
